# Beyond Performance: Cognitive Overload and Related Cognitive, Psychophysiological, and Performance States in Competitive Esports—A Scoping Review

**DOI:** 10.3390/medsci14030395

**Published:** 2026-07-15

**Authors:** Maciej Lachowicz, Dariusz Jamro, Anna Zawadka, Zofia Tomala, Grzegorz Żurek

**Affiliations:** 1 Department of Fundamentals of Physical Therapy and Occupational Therapy, Wroclaw University of Health and Sport Sciences, 51-612 Wroclaw, Poland; 2 Institute of Health Studies, University of Lower Silesia, 53-611 Wrocław, Poland; anna.zawadka@ideis.pl; 3 Department of Physical Education and Sport, General Tadeusz Kosciuszko Military University of Land Forces, 51-147 Wrocław, Poland; 4 Department of Biological Principles of Physical Activity, Wroclaw University of Health and Sport Sciences, 51-612 Wrocław, Poland; zosia.tomala@gmail.com (Z.T.); grzegorz.zurek@awf.wroc.pl (G.Ż.)

**Keywords:** esports, cognitive overload, mental workload, psychophysiology, human–computer interaction, performance, scoping review

## Abstract

**Background:** Competitive esports represents a rapidly developing digital performance environment in which players must regulate attention, affect, psychophysiological activation, and performance under dynamic, high-demand conditions. Although cognitive overload and related states are increasingly relevant to esports research, these constructs have not been consistently defined, operationalized, or measured. **Objective:** This scoping review aimed to map empirical evidence on cognitive overload and related states in competitive esports, with emphasis on cognitive demands, psychophysiological and neurophysiological markers, coping and recovery approaches, game genres, and performance outcomes. **Methods:** The review followed Joanna Briggs Institute guidance and PRISMA-ScR reporting principles. Searches were conducted in Scopus, PubMed, Web of Science, and EBSCOhost on 5 May 2026. Eligible studies were peer-reviewed empirical articles involving ranked, collegiate, semi-professional, professional, elite, or otherwise competitive esports players and included at least one objective or semi-objective indicator relevant to overload-related states. **Results:** Forty-eight studies were included. Evidence was distributed across in-game performance, stress and arousal, cardiometabolic load, mental workload, sleep and recovery, perceptual-cognitive processing, neurophysiological markers, and intervention-oriented approaches. Heart rate (HR), heart rate variability (HRV), eye-tracking, sleep or wearable measures, electroencephalography (EEG), event-related potential (ERP), cortisol, pupil diameter, electrodermal activity (EDA), cognitive tasks, and in-game metrics were used across the literature. However, these measures were rarely integrated into unified, multimodal models. **Conclusions:** Cognitive overload in esports is best understood as a dynamic, multidimensional state emerging from interactions between game demands, competitive context, player regulation, and performance consequences. Future research should develop genre-sensitive, multimodal, and time-synchronized models that distinguish workload, stress, fatigue, arousal, tilt, and performance decline.

## 1. Introduction

### 1.1. Background

Competitive esports can be understood as a high-demand form of human–computer interaction in which performance emerges from the continuous coordination of cognitive, affective, motor, and social processes [1,2]. Unlike casual gaming, competitive play is organized through visible systems of evaluation, including ranked ladders, matchmaking ratings, team structures, tournament formats, and win–loss outcomes [3,4]. As a result, the digital environment is not merely a platform for play, but a performance ecology in which players’ behaviour and psychophysiological responses are shaped by the evolving game state, the interface, opponents, teammates, and perceived competitive stakes [5,6]. Although esports has expanded rapidly during the twenty-first century, its relevance for psychological research lies less in its commercial growth than in the conditions under which players are required to perform. Competitive players must process rapidly changing information, make time-sensitive decisions, and maintain precise perceptual–motor control while adapting to game-specific performance demands and competitive pressure [7,8].

These demands are not confined to professional arenas or major tournaments. They may also emerge in ranked ladders, collegiate esports, team scrimmages, semi-professional competition, and structured practice, where repeated win–loss feedback, teammate dependence, visible progression systems, and critical in-game moments can make routine matches psychologically consequential. Research on competitive esports athletes has shown that performance-related, teammate-related, and critical-moment stressors are central features of competitive play, while studies in collegiate tournament settings indicate that esports competition can elicit measurable physiological and perceptual stress responses [9,10].

Player-generated terms such as tilt and ELO Hell suggest that competitive gaming communities have developed their own vocabulary for emotionally charged performance states. Although these terms did not originate from formal psychological theory, they capture experiences that players themselves perceive as central to ranked and competitive play, including loss of emotional control, perceived unfairness, difficulty recovering from negative in-game events, and performance breakdown. Recent empirical work has begun to formalize these community-derived concepts, linking tilt with emotion regulation and impaired gameplay, and ELO Hell with motivated bias in ranked environments [11,12].

Esports therefore provides a valuable context for studying human behaviour in interactive digital environments. It combines features that are useful for cognitive and performance research, such as repeated task exposure, rapid feedback, quantifiable performance, and objective behavioural traces, with the ecological validity of real competition, uncertainty, social evaluation, and meaningful performance consequences. This dual character positions esports as a bridge between laboratory-based research on human performance and naturalistic research on behaviour in computer-mediated environments [13,14].

### 1.2. Cognitive Overload in Esports

Cognitive overload can be understood as a state in which task demands approach or exceed the cognitive, attentional, or regulatory resources available to the individual, leading to reduced processing efficiency, compensatory effort, or performance disruption. Importantly, overload is not equivalent to high workload: high workload may remain adaptive when sufficient resources and regulation are available, whereas overload implies a mismatch between demands and capacity [15,16]. This distinction is particularly important in esports, where demanding play may reflect effective engagement, motivated effort, or adaptive challenge rather than impairment. Cognitive overload should therefore be understood not simply as “more demand,” but as a breakdown or destabilization of the balance between task demands, available resources, regulatory capacity, and performance efficiency.

In esports, such overload is unlikely to arise from a single source. Rather, it may develop when players must simultaneously process rapidly changing information, maintain situational awareness, coordinate perception and action, make time-sensitive decisions, and sustain performance across repeated or prolonged episodes of play [17,18]. Esports provides a distinctive context for studying these processes as competitive games combine dynamic visual displays, flexible allocation of attention, rapid feedback, and precise time-constrained motor control [19]. In this respect, esports resembles other high-demand performance environments in which efficient responding must be maintained under increasing stimulus density, uncertainty, and time pressure [20]. Within this demand structure, cognitive load and mental workload are the closest constructs to cognitive overload, as they describe the amount of processing and attentional regulation required by the task. Overload may be expected when these demands can no longer be regulated efficiently with the resources available to the player. The structure of demands varies across game genres rather than reflecting a single cognitive profile of esports performance. In multiplayer online battle arena games, players must integrate spatial, temporal, and team-level information over an evolving match, whereas shooter-based games tend to place greater emphasis on rapid target detection, inhibitory control, reaction speed, and sensorimotor precision. Other genres, including fighting and sport-simulation games, may impose different combinations of anticipation, timing, spatial positioning, and action selection. This genre specificity is important as cognitive overload is likely to reflect the demands of a particular digital task ecology rather than esports participation in general [21,22]. Research on multiplayer online battle arena (MOBA) games expertise supports this view, as professional players may differ from less experienced players not only in behavioural performance, but also in neural and game-specific indicators of expertise [23]. At the same time, the definition of elite or high-level esports samples remains inconsistent, and recent scoping work has shown that studies vary in how they classify player level, professional status, competitive achievement, and domain experience [24]. Player expertise should therefore be treated as a contextual moderator of overload-related processes rather than as a simple demographic descriptor.

Accordingly, cognitive overload in esports should not be conceptualized as a uniform response to “gaming” in general [23]. The cognitive and psychophysiological profile of a ranked League of Legends match may differ substantially from that of a Counter-Strike clutch situation, a Rocket League overtime sequence, a FIFA match, or a first-person shooter (FPS) aiming task [25]. Even within the same game, the relevant demands may fluctuate depending on match phase, score, role, opponent level, communication quality, fatigue, recent errors, or perceived match importance. Thus, cognitive overload in esports is best understood as a dynamic state arising from the interaction between player characteristics, game genre, competitive context, and moment-to-moment in-game events [5,26].

This dynamic character explains why stress, arousal, affective states, and emotion regulation are relevant to the present review, while also requiring conceptual caution. Competitive esports is not a neutral cognitive task. It is performed under conditions of evaluation, uncertainty, social comparison, and emotional salience. Emotional states may alter how players appraise a situation, allocate attention, mobilize effort, and recover from mistakes. Experimental work in an esports-like FIFA context suggests that emotions with stronger approach tendencies may facilitate performance through challenge-related cognitive and cardiovascular responses [27]. This indicates that physiological activation cannot be interpreted automatically as overload. It may reflect adaptive engagement, motivation, or challenge, particularly when the player remains able to regulate performance effectively. Similarly, research on emotions and emotion regulation in esports shows that emotional experiences influence performance and that players actively attempt to regulate these states during competitive play [28]. Stress and arousal are therefore best understood as adjacent affective and psychophysiological correlates. They may accompany high cognitive demand, intensify it, or alter its consequences, but they do not define cognitive overload on their own.

Fatigue, sleepiness, and insufficient recovery occupy a different position in this framework. They are not direct manifestations of cognitive overload, but they may reduce the player’s available cognitive and regulatory resources. Under rested and well-regulated conditions, a demanding match may remain manageable; under fatigue or poor recovery, similar task demands may become inefficient, frustrating, or performance-disruptive. This is especially relevant in esports, where long practice schedules, sedentary play, sleep disturbance, burnout risk, and health-related concerns are part of the broader performance environment [29]. These factors may lower the threshold at which workload becomes overload and may also influence the interpretation of physiological markers, subjective strain, and performance decline.

The same caution applies to interventions and strategies framed as cognitive support, recovery enhancement, or performance optimization. Esports players and organizations may seek methods to improve attention, regulate arousal, accelerate recovery, or protect performance under pressure, yet the evidence supporting such strategies is uneven. Approaches such as sleep extension, napping, and structured recovery management are conceptually aligned with the demand–resource framework as they target the restoration of cognitive and regulatory resources [30]. By contrast, strategies aimed primarily at increasing activation, wakefulness, or perceived focus require greater caution, including high-dose caffeine or energy drinks, commercial nootropics, auditory or neurostimulation tools, and the nonmedical use of prescription stimulants such as mixed amphetamine salts, methylphenidate, or modafinil [31,32]. Evidence from home-use binaural beats brain stimulation shows that interventions promoted as cognitive support may fail to improve performance and may even impair cognitive outcomes [33], while broader work on non-invasive brain stimulation and computerized brain training suggests that cognitive-enhancement effects in healthy adults are often inconsistent, task-specific, or limited in their transfer to real-world functioning [34,35]. Pharmacological enhancement shows a similar need for caution: systematic and meta-analytic work on modafinil, methylphenidate, and d-amphetamine suggests that effects are often domain-specific, modest, dependent on sleep loss, baseline state, task complexity, and outcome selection and may not translate straightforwardly into complex real-world performance [36,37]. Therefore, reducing cognitive overload should not be equated with simply increasing stimulation, wakefulness, or perceived focus; it requires identifying whether the limiting factor is excessive task demand, insufficient recovery, poor emotion regulation, maladaptive arousal, lack of expertise, or a mismatch between the intervention and the specific game context. Existing esports research has approached this phenomenon through several related constructs, including mental workload, stress, arousal, fatigue, sleepiness, attentional control, tilt, performance decline, and psychophysiological regulation [38,39]. These constructs are related but not interchangeable. Mental workload describes the demands placed on limited cognitive resources, whereas stress and arousal refer to psychophysiological responses to challenge, threat, or evaluation. Fatigue and sleep loss may further reduce cognitive efficiency, particularly in sustained attention and executive control, while performance decline represents a behavioural manifestation of these interacting processes [18,40]. Tilt is especially relevant in esports as it captures a player-recognized state of emotional dysregulation, perceived loss of control, difficulty recovering from mistakes, and impaired gameplay. However, tilt may arise from several interacting sources, including frustration, teammate conflict, repeated failure, perceived unfairness, fatigue, or poor coping. It should therefore be interpreted as a possible functional consequence of overload-related processes rather than as a direct measure of cognitive overload itself.

**Figure 1:**
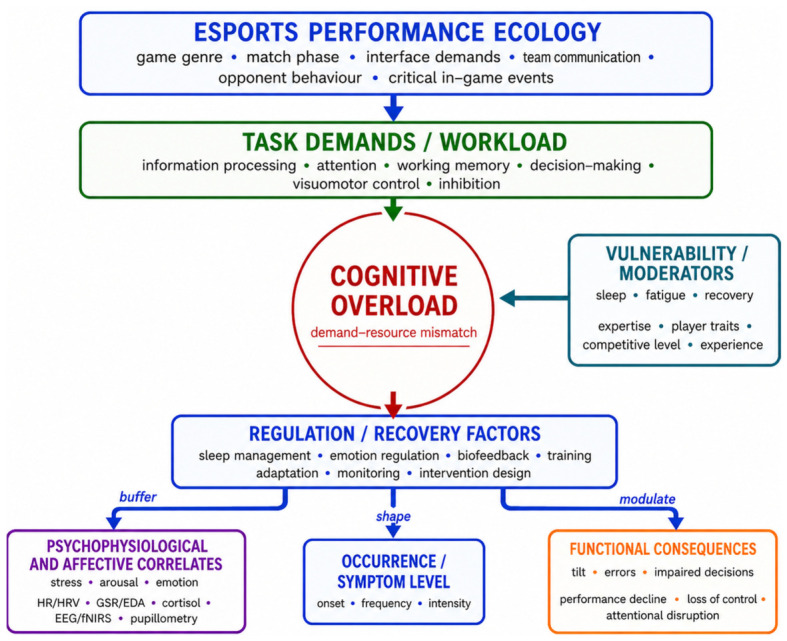
Demand–resource–regulation framework of cognitive overload in competitive esports.

Cognitive overload can therefore be treated as an umbrella construct linking these domains, particularly when subjective states are accompanied by objective or semi-objective indicators of workload, arousal, fatigue, or performance change [41]. To make this umbrella construct analytically useful, its surrounding domains should be organized according to their functional role. As shown in Figure 1, esports performance ecology and task demands provide the context in which cognitive load and mental workload are placed on the player. Cognitive overload is then conceptualized as a demand–resource mismatch, influenced by vulnerability and moderating factors such as sleep, fatigue, recovery status, expertise, competitive level, and player characteristics. Importantly, regulation and recovery factors may shape how overload is expressed: they may buffer psychophysiological and affective correlates, influence the occurrence and intensity of overload-related symptoms, and modulate functional consequences such as tilt, attentional disruption, impaired decision-making, errors, and performance decline. This structure allows cognitive overload to be approached as an integrative construct while avoiding the assumption that every demanding, emotional, physiological, or performance-disruptive state is itself cognitive overload.

### 1.3. Rationale for the Scoping Review

The literature on cognitive overload and related states in esports is growing but remains conceptually and methodologically fragmented. Existing research has examined physical, psychological, health-related, and performance-related variables, often across different disciplinary traditions and with varying assumptions about what constitutes relevant evidence for esports performance [42]. At the measurement level, studies increasingly combine heterogeneous data streams, including physiological, affective, behavioural, video-based, cognitive-performance, and in-game indicators, reflecting the multimodal nature of esports as a performance environment [43]. In addition to observational and measurement-oriented research, esports has also motivated intervention-oriented work targeting cognitive and performance-related functions. These approaches include immersive virtual reality interventions aimed at improving attention, visuospatial memory, reaction time, and eye–hand coordination in e-athletes [44,45]. Such studies are relevant to the present review when they inform coping, recovery, training, or performance-related responses to cognitive-overload-related demands.

This conceptual and methodological diversity is valuable, but it also creates challenges for interpretation. Related states are described through partially overlapping terminology, including mental workload, cognitive load, fatigue, stress, arousal, tilt, and performance breakdown. Measurement approaches also differ substantially, with cognitive-overload-related states inferred from physiological, neurophysiological, ocular, behavioural, subjective, sleep-related, and in-game performance indicators. As a result, it remains unclear how cognitive overload has been operationalized in esports research, which markers have been used most frequently, which game genres and competitive settings have been studied, and where the most important evidence gaps remain. Additionally, as cognitive overload has rarely been operationalized directly in esports research, this review intentionally mapped adjacent constructs only when they could plausibly inform the assessment, interpretation, or behavioural consequences of overload-related states.

A scoping review is therefore appropriate as the aim is not to estimate a single pooled effect, but to map the extent, range, and nature of the available evidence in an emerging and methodologically heterogeneous field. This approach is particularly suitable when the purpose is to identify knowledge gaps, clarify concepts, examine how research has been conducted, and describe the scope of an evidence base [46]. A further rationale for this review is the need to distinguish competitive esports from casual gaming and from research focused primarily on problematic gaming or gaming disorder. While gaming disorder research is important, it addresses different questions, including the assessment of disordered gaming symptoms and the applicability of clinical frameworks to esports populations [47]. The present review focuses specifically on competitive esports contexts in which players are engaged in ranked, collegiate, semi-professional, professional, or otherwise structured competitive play. This distinction is necessary as the psychological meaning of play changes when performance is evaluated, ranked, socially visible, or tied to team and competitive outcomes.

### 1.4. Objectives and Research Questions

The objective of this scoping review was to map the existing empirical literature on cognitive overload and related constructs in competitive esports, with particular attention to cognitive demands, psychophysiological and neurophysiological markers, coping and recovery approaches, game genres, and performance outcomes. Specifically, the review aimed to identify how cognitive-overload-related states have been conceptualized and measured, which objective or semi-objective indicators have been used, which esports genres and competitive contexts have been examined, and what methodological and conceptual gaps remain.

The review was guided by the following research questions:

1. What cognitive demands and cognitive-overload-related constructs have been investigated in competitive esports contexts?
2. Which psychophysiological, physiological, neurophysiological, cognitive-performance, behavioural, sleep-related, or in-game performance indicators have been used to assess cognitive overload or related states in esports players?
3. Which game genres and competitive contexts have been examined in relation to cognitive overload, stress, fatigue, arousal, recovery, coping, and performance?
4. What coping, recovery, or intervention approaches have been investigated in relation to cognitive overload or performance-related strain in esports?
5. What evidence gaps remain in the assessment and interpretation of cognitive overload in competitive esports?

## 2. Methods

### 2.1. Study Design

This study was conducted as a scoping review to systematically map the extent, range, and characteristics of the literature concerning cognitive overload and related constructs in esports. A scoping review design was considered appropriate as the objective was not to determine intervention effectiveness or estimate pooled effect sizes, but to identify the types of evidence available, clarify how relevant constructs have been operationalized, examine methodological approaches used in the field, and identify knowledge gaps. The review was designed in accordance with the updated Joanna Briggs Institute (JBI) methodological guidance for scoping reviews and reported in line with the PRISMA extension for Scoping Reviews [48,49,50] (Supplementary File S2).

The review process comprised formulation of the research questions, development of eligibility criteria using the Population–Concept–Context framework, systematic database searching, study selection, data charting, descriptive evidence mapping, and narrative synthesis.

### 2.2. Protocol and Registration

A review protocol was deposited on the Open Science Framework before completion of full-text screening, data charting, and evidence synthesis. The protocol is currently under embargo and can be made available to editors and reviewers on request where permitted; it will be made public following embargo release. The protocol specified the rationale, objectives, review questions, eligibility criteria, information sources, search strategy, study selection procedure, data charting plan, and synthesis approach. Any deviations from the protocol are described below in the relevant Methods subsections.

The manuscript title was refined from the protocol title to emphasize that the review mapped cognitive overload and related states, which is consistent with the protocol’s Concept definition and research questions. No changes were made to the review objectives, eligibility criteria, or search strategy as a result of this title refinement.

Compared with the protocol, the review questions were editorially consolidated in the final manuscript to reduce redundancy. The protocol question concerning cognitive and in-game performance outcomes was incorporated into the questions addressing objective/semi-objective indicators, game genres and competitive contexts, and coping/intervention approaches. This consolidation did not change the review objective, eligibility criteria, search strategy, screening decisions, or data charting framework.

### 2.3. Eligibility Criteria/PCC Framework

Eligibility criteria were structured using the PCC framework presented in Table 1 below.

Studies were eligible for inclusion if they met all of the following criteria:

- they were peer-reviewed original empirical studies;
- were available in English;
- had full text available;
- involved esports players with at least ranked, competitive, collegiate, semi-professional, professional, or elite status;
- examined an esports or competitive gaming context;
- and included at least one objective or semi-objective indicator relevant to cognitive overload or related psychophysiological, cognitive, behavioural, sleep-related, or performance-based states.

In this review, objective indicators were defined as instrument-, software-, or system-derived measures that did not rely primarily on self-report, including physiological, psychophysiological, neurophysiological, ocular, endocrine, sleep-related, behavioural, cognitive-performance, and in-game telemetry measures. Semi-objective indicators were defined as structured or system-derived measures that were not purely subjective but still depended on task context, author-defined thresholds, game-specific algorithms, wearable algorithms, or performance-classification procedures. Examples included rank or matchmaking rating, game statistics, performance-decline labels, expert–novice classifications, wearable-derived sleep summaries, and standardized task-performance outcomes. Subjective measures were defined as self-reported questionnaires, ratings, or interviews and were not sufficient for inclusion unless accompanied by at least one objective or semi-objective indicator.

To avoid overly broad inclusion of general esports performance studies, objective or semi-objective indicators were considered relevant only when they were explicitly linked to cognitive-overload-related constructs, such as mental workload, cognitive load, stress, arousal, fatigue, sleepiness, attentional control, recovery, psychophysiological regulation, performance decline, or performance under competitive strain. Studies were not included solely as they reported game statistics or physiological values; these indicators had to be interpreted in relation to cognitive, psychophysiological, behavioural, sleep-related, or performance-regulation processes relevant to overload or adjacent states.

Competitive status was determined from author-reported information, including official rank, participation in organized tournaments or leagues, collegiate esports membership, semi-professional or professional status, elite classification, regular structured team-based competitive practice, or participation in ranked or otherwise structured competitive game modes. In this review, recreational competitive players were distinguished from casual or purely recreational gamers. This category referred to players who engaged in ranked, matchmaking-based, ladder-based, or otherwise competitive modes, but who were not described in the original studies as collegiate, semi-professional, professional, or elite players, and who did not necessarily participate in organized leagues or tournaments. A universal threshold based on minimum rank, league level, tournament participation, team membership, or training frequency was not imposed, as ranking systems and competitive structures differ substantially across game titles, platforms, regions, and study designs, and such details were often incompletely reported in the original articles. Studies involving only casual or recreational gaming without evidence of ranked, competitive, structured performance context, or esports-specific relevance were excluded.

Studies were also excluded if they focused exclusively on subjective psychological, stress, fatigue, coping, sleep, or mood outcomes without any objective or semi-objective indicator; gaming disorder, gaming addiction, problematic gaming, or internet gaming disorder as the primary focus; aggression, violence, gambling, education, rehabilitation, spectatorship, advertising, marketing, or business aspects; non-original publications; conference abstracts, proceedings, patents, preprints, or other non-peer-reviewed materials; or technical, tool-development, or dataset papers without eligible empirical analysis of esports players.

The review was restricted to peer-reviewed journal articles to ensure a minimum level of methodological reporting and scientific scrutiny.

### 2.4. Information Sources and Search Strategy

The literature search was conducted in four electronic databases: Scopus, PubMed, Web of Science, and EBSCOhost. Searches were conducted on 5 May 2026, with no restrictions on publication date. Where database functionality allowed, searches were limited to English-language records. The number of records identified from each database was as follows: Scopus, 364 records; Web of Science, 256 records; PubMed, 121 records; and EBSCOhost, 21 records. In total, 762 records were identified before duplicate removal.

The EBSCOhost search was conducted across all databases available through the institutional subscription at the time of searching. The searched EBSCOhost databases included multidisciplinary, biomedical, sport science, education, business, and information science collections. The complete list of EBSCOhost databases searched on 5 May 2026 is provided in Supplementary Table S4. The EBSCOhost strategy was simplified as broad free-text combinations across all available EBSCOhost databases generated excessive noise and database-specific query limitations. Core marker families were retained to preserve conceptual coverage while maintaining search feasibility.

The search strategy was developed to identify studies situated at the intersection of esports, cognitive-overload-related constructs, psychophysiological and neurophysiological markers, cognitive-performance indicators, coping or recovery approaches, game genres, and performance outcomes. Search terms combined esports-related terminology with terms referring to cognitive and psychological constructs, physiological and neurophysiological indicators, performance-related outcomes, and game-genre descriptors.

The strategy was adapted to the syntax and indexing structure of each database. Full electronic search strings for all databases are provided in Supplementary File S1. Search strings were adapted to database-specific syntax and practical search constraints; therefore, the exact number of title-specific and marker-specific terms varied across databases.

Although the protocol allowed supplementary searching through reference-list screening and citation chasing, no additional supplementary searching was conducted. The final review was therefore based exclusively on database searches in Scopus, PubMed, Web of Science, and EBSCOhost. This deviation from the protocol was made to preserve a reproducible database-based search strategy and is reflected in the PRISMA flow diagram, which reports records identified through databases only.

### 2.5. Study Selection

All records were screened independently by two reviewers using predefined eligibility criteria based on the Population–Concept–Context framework. Before formal screening, the reviewers completed a calibration exercise to ensure consistent application of the inclusion and exclusion criteria. Initial discrepancies were primarily related to publication type, particularly the handling of reviews, conference proceedings, and other non-original or non-peer-reviewed records, as well as the interpretation of competitive status and eligible objective or semi-objective indicators. After discussion, the eligibility criteria and operational definitions were clarified, and both reviewers proceeded with calibrated title-and-abstract screening.

Inter-reviewer agreement was assessed at the title-and-abstract screening stage using percentage agreement and Cohen’s kappa. For this calculation, “include” and “maybe” decisions were coded as “retain,” whereas “exclude” decisions were coded as “exclude,” reflecting the conservative screening approach used in the review. After calibration, inter-reviewer agreement was 81.2%, with Cohen’s κ = 0.42. Records judged as ambiguous by either reviewer, as well as records with discordant reviewer decisions, were retained for full-text assessment to minimize premature exclusion. The moderate kappa value may reflect the conceptual breadth of the review topic and the difficulty of consistently distinguishing studies directly addressing cognitive overload from studies addressing adjacent constructs.

Full-text articles were then assessed independently by two reviewers. Disagreements at the full-text stage were resolved through discussion and consensus, with consultation of a third reviewer when consensus could not be reached. Before final synthesis, the included studies were re-audited against the mandatory eligibility criteria, particularly competitive status, the presence of an eligible objective or semi-objective indicator, and relevance to cognitive overload or related states. Studies that did not meet the objective or semi-objective indicator requirement, or that represented dataset-descriptor articles without eligible primary empirical analysis, were excluded and documented in the excluded-studies table.

All records identified through database searching were imported into Rayyan for duplicate removal and screening. Rayyan was used to organize records, manage screening decisions, and support documentation of the study selection process [51]. After removal of 284 duplicate records, 478 records remained for title and abstract screening. The study selection process is reported in the Results section and presented using a PRISMA-style flow diagram adapted for a scoping review.

### 2.6. Data Charting

A standardized data charting form was developed on the basis of the review questions, eligibility criteria, and Population–Concept–Context framework. The form was designed to capture bibliographic, methodological, population-related, esports-specific, measurement-related, and outcome-related information from each included study. Before final extraction, the charting form was piloted on a subset of included studies representing different study designs, game genres, and marker types. The pilot exercise was used to refine category definitions, clarify coding rules, and ensure that the form captured the heterogeneity of esports contexts, measurement approaches, and overload-related constructs.

The following data items were charted: bibliographic information; country or region; study design; study context; sample size; participant age and participant sex or gender as reported by the original study authors; player level and competitive status; basis for competitive status; game title and genre; laboratory, field, training, online, home-based, or tournament setting; cognitive-overload-related construct or domain; eligibility-confirming objective or semi-objective indicator; physiological, psychophysiological, neurophysiological, sleep-related, cognitive-performance, behavioural, and in-game performance markers; subjective measures where present; intervention or exposure characteristics; main findings; relevance to the review questions; and methodological limitations or notes.

Data charting was conducted using the standardized form and then verified by a second reviewer. The verification focused on fields directly affecting eligibility and evidence mapping, including competitive status, objective/semi-objective indicator, game title and genre, study design, marker category, cognitive-overload-related domain, and performance or cognitive outcome category. Discrepancies were resolved through discussion and consensus; when consensus could not be reached, a third reviewer was consulted. Data charting was iterative, and refinements to category definitions were documented during extraction. The final charting table is provided in Supplementary Table S1.

### 2.7. Data Synthesis

Data synthesis followed a descriptive and mapping-oriented approach. Because the objective of the review was to map heterogeneous evidence rather than to synthesize effect estimates, no meta-analysis was conducted.

First, descriptive numerical summaries were generated to characterize the included studies by publication year, country or region, study design, player population, competitive level, game title, game genre, study context, marker type, and outcome domain. Second, evidence was mapped across cognitive-overload-related domains, including stress and arousal, mental workload, fatigue, sleep and recovery, visual attention, neurophysiological indicators, cognitive-performance outcomes, in-game performance outcomes, and intervention or coping-related approaches. Third, a narrative synthesis was conducted to organize the evidence around the review questions and identify recurring methodological patterns, conceptual emphases, and evidence gaps.

The narrative synthesis was structured around the following thematic areas: cognitive demands and mental workload in esports; psychophysiological and neurophysiological markers; sleep, fatigue, recovery, and prolonged gameplay; game genres and competitive contexts; coping, intervention, and performance-related outcomes; and methodological limitations and evidence gaps.

Formal risk-of-bias assessment or methodological quality appraisal was not conducted. This decision is consistent with scoping review guidance, in which critical appraisal is optional and generally not required when the purpose is to map the available evidence rather than to provide a synthesized answer to a narrowly defined question [49,50].

### 2.8. Ethical Considerations

This review was based exclusively on previously published literature and did not involve the collection of new data from human participants. Therefore, ethics committee approval was not required.

The review protocol was registered on OSF to support transparency and reproducibility. Search strategies, full-text exclusion reasons, and the final data charting table will be made available as Supplementary Materials or through the OSF project repository where permitted. Copyrighted full-text articles will not be shared in the repository.

## 3. Results

### 3.1. Study Selection Results

**Figure 2:**
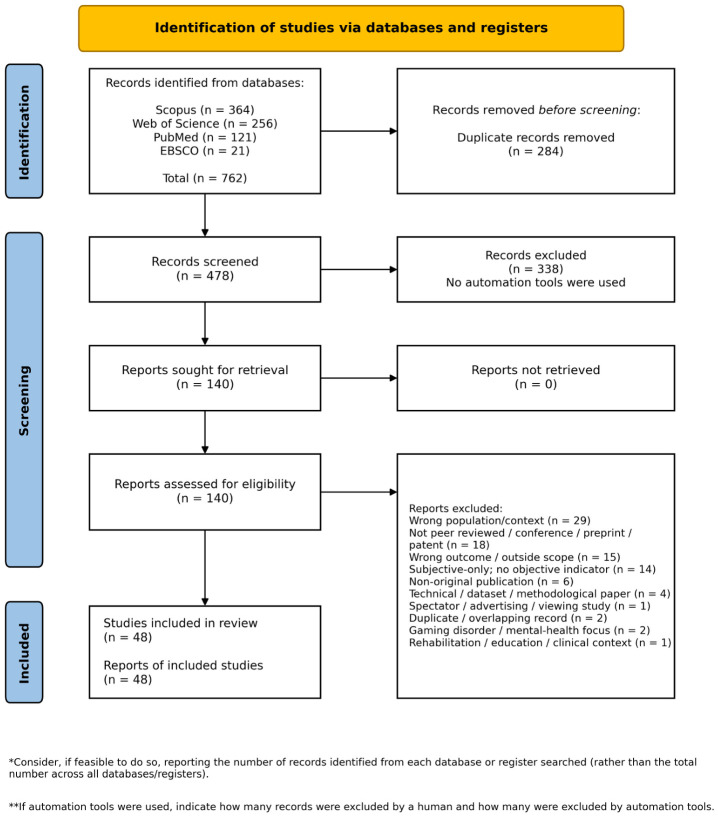
PRISMA-style flow diagram of the study selection process.

The database search identified 762 records: 364 from Scopus, 256 from Web of Science, 121 from PubMed, and 21 from EBSCOhost. After removal of 284 duplicate records, 478 records remained for title and abstract screening. Of these, 338 records were excluded. The remaining 140 studies were retrieved and assessed for eligibility in full text. Following full-text assessment, 92 studies were excluded with documented reasons, and 48 studies were included in the final scoping review. The study selection process is presented in Figure 2. A complete list of full-text exclusions with reasons is provided in Supplementary Table S3. All included studies are listed in Supplementary Table S1 and are marked with an asterisk in the reference list.

The most frequent reasons for exclusion at the full-text stage were wrong population or context, non-peer-reviewed or conference-type publication status, wrong outcome or outside-scope focus, subjective-only outcomes without objective or semi-objective indicators, and non-original publication type.

### 3.2. Characteristics of Included Studies

The 48 included studies were published between 2016 and 2026, with a marked increase in publications in recent years. Most studies were published from 2021 onward (*n* = 42; 87.5%), and the largest number of publications occurred in 2024 (*n* = 15; 31.3%). Earlier studies were comparatively sparse, with one study published in 2016, one in 2018, one in 2019, and three in 2020. The included studies represented a geographically diverse but unevenly distributed body of literature. The most frequently represented countries or regions were the United States (*n* = 7), Japan (*n* = 6), China (*n* = 7), Germany (*n* = 4), Australia (*n* = 3), and Spain (*n* = 3). Additional studies were conducted in, or involved data from, countries including Poland, Switzerland, Ukraine, Brazil/Portugal, Norway, Russia, South Korea, Ireland, Taiwan, and the United Kingdom. Several studies involved multi-country samples or used publicly available online data.

Study designs were heterogeneous and included experimental studies, cross-sectional comparisons, observational field studies, longitudinal monitoring studies, randomized or controlled interventions, secondary analyses of existing datasets, machine-learning studies, and psychophysiological laboratory protocols. The designs reflected the exploratory and multidisciplinary character of the field, with studies drawing on sport science, psychology, neuroscience, human–computer interaction, sleep science, nutrition, and performance analysis.

Sample sizes varied considerably. Among studies reporting participant samples, the smallest studies involved single professional teams or pilot samples, whereas the largest involved survey, intervention, or dataset-based samples exceeding 250 participants. Several studies were based on small groups of elite or professional esports players, which is relevant given the difficulty of recruiting high-level competitive players. However, many studies relied on male-only or predominantly male samples, indicating limited representation of female esports athletes.

The included studies examined a range of competitive levels, including ranked players, collegiate esports players, semi-professional players, professional players, elite esports athletes, high-skill players, and expert–novice comparison groups. Competitive status was typically operationalized through official rank, professional or semi-professional affiliation, collegiate team membership, tournament participation, or author-defined expertise categories.

A shortened overview of the included studies, including bibliographic characteristics, study design, sample, player level, game title, game genre, and main markers or outcomes, is provided in Table 2. The full version of the table is available in Supplementary Table S1.

### 3.3. Cognitive-Overload-Related Domains

The evidence was organized into non-mutually exclusive domains, meaning that a single study could contribute to more than one domain. The most frequently represented domain was in-game performance, practice, and expertise outcomes (*n* = 37), followed by stress, arousal, and autonomic regulation (*n* = 29). Cardiometabolic and physiological load was represented in 24 studies, while mental workload, cognitive load, and performance decline appeared in 20 studies. Cardiometabolic and general physiological indicators were treated as contextual markers of gameplay- or competition-related strain rather than as direct indicators of cognitive overload. Sleep, fatigue, and recovery were represented in 19 studies. Visual attention, gaze, and perceptual-cognitive processing were identified in 15 studies, and neurophysiological or cortical markers were present in 15 studies. Intervention, coping, and regulation strategies were addressed in 12 studies. These distributions indicate that the included literature covered several partially overlapping evidence domains, with no single domain accounting for the majority of included studies. Across the included studies, cognitive overload was not consistently defined as a single construct. Instead, overload-related evidence was reported through a network of related domains, including mental workload, stress, arousal, fatigue, sleep disruption, cognitive decline, attentional control, performance breakdown, and psychophysiological regulation. This conceptual heterogeneity is characteristic of an emerging field and supports the need for evidence mapping rather than pooled quantitative synthesis.

A shortened evidence map of cognitive-overload-related domains, common markers, typical outcomes, and interpretation for the review is provided in Table 3. The full version is available in Supplementary Table S2.

### 3.4. Psychophysiological and Neurophysiological Markers

**Figure 3:**
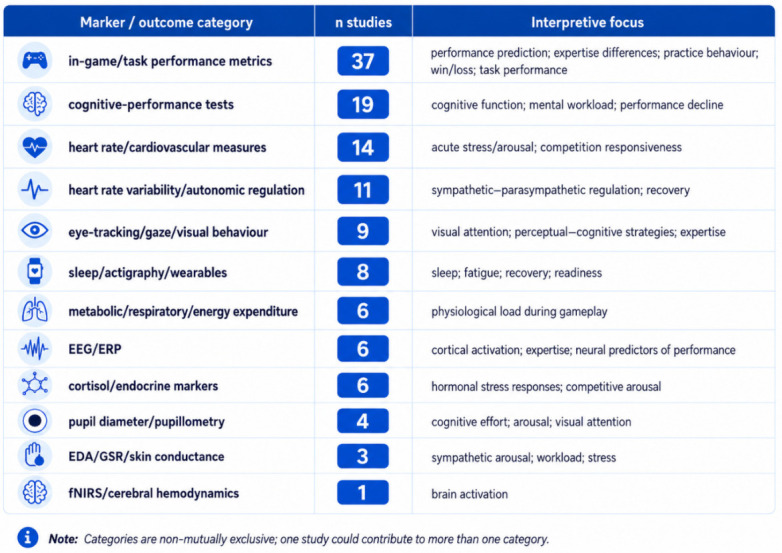
Frequency of objective and semi-objective marker use across included studies.

Psychophysiological and physiological markers were central to a substantial proportion of the included literature. As presented in Figure 3, HR and HRV were among the most frequently used indicators, particularly in studies examining acute stress responses, autonomic regulation, victory versus defeat, competitive pressure, and gameplay-induced physiological load. Heart rate was used as a general indicator of cardiovascular activation, whereas HRV indices were commonly interpreted in relation to sympathetic–parasympathetic regulation.

Endocrine and biochemical markers were less frequent but appeared in studies examining stress physiology and competitive arousal. Cortisol was the most common endocrine marker, particularly in studies assessing pre- and post-competition responses or the relationship between anxiety, arousal, and performance. Several studies also included blood pressure, pulse-wave velocity, glucose, lactate, oxygen consumption, carbon dioxide production, respiratory exchange ratio, or energy expenditure, thereby extending the evidence base beyond autonomic markers alone.

Neurophysiological approaches included EEG, event-related potentials, spectral power analysis, resting-state EEG complexity, and fNIRS. These measures were used mainly to characterize expertise, cortical activation, cognitive control, neural predictors of performance, or intervention-related changes. Although these studies provide promising evidence that neural markers may distinguish expertise levels or predict performance-related states, the methodological approaches were heterogeneous and often based on small samples.

Eye-tracking and gaze-related measures formed another prominent measurement cluster. These studies examined fixation count, fixation duration, gaze distribution, quiet-eye metrics, gaze strategies, scan patterns, pupil diameter, visual search, multiple-object tracking, and short-term visual memory. The findings suggest that perceptual-cognitive processing is a key component of esports performance, although expertise-related advantages appear to depend strongly on the task, game genre, and degree of ecological validity.

### 3.5. Game Genres and Competitive Contexts

The included studies covered multiple esports titles and game genres, with multiplayer online battle arena and shooter-based games being the most frequently represented. In non-mutually exclusive genre coding, first-person shooter, tactical shooter, or hero-shooter contexts appeared in 23 studies, while multiplayer online battle arena games appeared in 22 studies. Sports simulation or virtual football games were represented in 5 studies, fighting games in four, Rocket League or vehicular soccer contexts in three, and FPS-like aiming tasks in three. Several studies included mixed or multiple game genres.

League of Legends and Counter-Strike/Counter-Strike: Global Offensive were the most common individual titles. League of Legends appeared in 19 studies, while Counter-Strike-related contexts appeared in 13 studies. Other titles included FIFA/eFootball, Valorant, Overwatch, Rocket League, Dota 2, Call of Duty, Rainbow Six Siege, PlayerUnknown’s Battlegrounds, and AimLab-based aiming tasks.

Study contexts ranged from controlled laboratory protocols to real or simulated competitive environments. Laboratory-based studies allowed standardized assessment of physiological, cognitive, and neurophysiological variables, whereas field and tournament-based studies provided greater ecological validity. Several studies used official matches, professional team data, local area network (LAN) events, training facilities, home-based protocols, or wearable monitoring across multiple days or weeks. This diversity indicates that esports research is developing across both controlled experimental and ecologically situated settings, but it also limits direct comparability across studies.

### 3.6. Sleep, Fatigue, Recovery, and Coping/Intervention Approaches

Sleep, fatigue, and recovery formed a substantial evidence cluster. Studies in this domain used actigraphy, wearable devices, sleep diaries, Oura- or Somnofy-derived sleep measures, psychomotor vigilance testing, reaction-time tasks, subjective sleepiness or fatigue ratings, HRV, and pupil diameter. The literature addressed delayed sleep timing, sleep duration, sleep quality, sleep deprivation, post-game arousal, recovery, mood, and cognitive performance.

Sleep-related studies generally indicated that sleep and recovery are relevant to esports performance and health, but the evidence remains heterogeneous. Some studies focused on professional or elite esports athletes, whereas others examined ranked or experienced players. Designs included cross-sectional sleep profiling, longitudinal sleep monitoring, sleep intervention, sleep extension feasibility protocols, and experimental sleep deprivation. Together, these studies suggest that sleep may influence cognitive readiness, autonomic regulation, mood, and performance-related functioning, although causal evidence remains limited.

Intervention and coping-related studies represented an emerging but diverse area. The included interventions and regulation strategies included biofeedback training, arousal reappraisal, mindset-based approaches, sleep counselling or sleep education, exercise interventions, energy drink consumption, nutrition/lifestyle assessment, and coping-related psychological measures. These studies targeted outcomes such as anxiety, challenge–threat appraisal, cognitive performance, gaze control, HRV, cerebral oxygenation, aiming performance, sleep, mood, and task accuracy.

Overall, the intervention evidence suggests growing interest in performance optimization and regulation of cognitive or psychophysiological load. However, the evidence base is still fragmented, with small samples, short intervention durations, varied outcome measures, and limited replication across games and competitive levels.

### 3.7. Performance Outcomes

Performance outcomes were the most frequently represented evidence domain. These outcomes included match result, win/loss status, kills/deaths/assists (KDA), game logs, game statistics, rank or matchmaking rating, task accuracy, shot time, completion time, reaction time, practice volume, performance-decline labels, and expert–novice classification. Some studies examined real in-game metrics derived from professional or competitive matches, whereas others relied on standardized esports-like tasks, laboratory-based performance tasks, or proxy indicators of expertise and practice behaviour.

Performance was commonly examined in relation to psychophysiological, cognitive, neurophysiological, or behavioural indicators. For example, included studies investigated whether autonomic regulation differed between winners and losers, whether gaze behaviour distinguished professional from non-professional players, whether electroencephalographic or cortical markers were associated with expertise or match outcomes, whether sleep deprivation affected in-game performance, and whether psychological or physiological interventions improved task performance. Despite the centrality of performance in the included literature, its operationalization varied substantially across studies. Some outcomes reflected actual competitive match performance, whereas others captured narrower task-based indicators, such as aiming accuracy, cognitive-task performance, reaction time, expertise classification, or practice behaviour. This variability limits direct comparison across studies, but it also illustrates the breadth of approaches used to assess performance-related states in esports research.

Across the mapped domains, the observed associations generally suggested that competitive pressure, match outcome, and high-demand gameplay were linked with increased autonomic, cardiovascular, endocrine, or broader psychophysiological activation. However, such activation could not be interpreted as cognitive overload on its own, as it may also reflect adaptive engagement, competitive arousal, or challenge-related responding. Expertise-related studies tended to indicate more efficient or game-specific perceptual-cognitive, gaze, behavioural, or neurophysiological patterns among higher-level players. Sleep, fatigue, and recovery studies generally suggested that insufficient sleep, delayed sleep timing, or sleep deprivation may be associated with reduced cognitive readiness, slower reaction time, mood changes, autonomic alterations, or poorer performance-related functioning. Intervention-oriented studies reported preliminary and mixed effects on arousal regulation, sleep, cognitive performance, gaze control, aiming performance, and psychophysiological markers. Overall, the direction of evidence supports the relevance of cognitive, physiological, sleep-related, and performance indicators for understanding overload-related states in esports, but the heterogeneity of study designs, markers, outcomes, and game contexts prevents stronger causal or marker-specific conclusions.

### 3.8. Evidence Gaps

Several evidence gaps were identified.

First, cognitive overload remains inconsistently defined and operationalized in esports research. Many studies examined related constructs such as stress, arousal, fatigue, workload, sleepiness, tilt, cognitive decline, or performance pressure, but few explicitly defined cognitive overload as a central construct.

Second, the literature is methodologically heterogeneous. Studies differ substantially in design, sample characteristics, game genre, competitive level, measurement protocol, and outcome definition. This heterogeneity supports the appropriateness of a scoping review approach but limits direct comparison and precludes quantitative synthesis.

Third, samples were frequently small, male-dominated, and drawn from specific competitive levels or single teams. Professional and elite players were represented, but access to these populations remains limited. Female esports athletes and mixed-gender competitive samples were underrepresented.

Fourth, many studies used laboratory or simulated esports tasks. Although such designs provide experimental control, they may not fully capture the dynamic, social, strategic, and high-pressure characteristics of real esports competition. Conversely, field and tournament-based studies offer greater ecological validity but often include less experimental control.

Fifth, marker integration remains limited. Although HR, HRV, EEG, eye-tracking, cortisol, sleep measures, cognitive tests, and in-game performance data have all been used, relatively few studies combined multiple modalities in a way that allows robust modelling of cognitive overload across physiological, cognitive, behavioural, and performance domains.

Finally, intervention evidence remains preliminary. Biofeedback, reappraisal, mindset, sleep, exercise, nutrition, and ergogenic strategies have been investigated, but replication is limited, and few studies have assessed long-term effects, transfer to real competition, or sustained performance outcomes. Future research should prioritize longitudinal, multimodal, ecologically valid, and game-specific designs that integrate objective markers with meaningful competitive performance outcomes.

## 4. Discussion

The principal contribution of this scoping review is to show that cognitive overload in esports has not yet been operationalized as a stable and consistently measured construct; rather, current evidence approaches it indirectly through overlapping domains of mental workload, stress and arousal, fatigue and recovery, perceptual-cognitive processing, and performance decline [52]. From a human–computer interaction perspective, this finding positions esports as a complex computer-mediated performance environment in which cognitive, affective, behavioural, and psychophysiological processes are continuously shaped by interaction with the game system, teammates, opponents, and competitive consequences [53].

### 4.1. Key Findings

This review included 48 empirical studies addressing cognitive-overload-related constructs in competitive esports contexts. The evidence base was broad, but conceptually and methodologically dispersed, with included studies distributed across performance, stress and arousal, cardiometabolic load, mental workload, sleep and recovery, perceptual-cognitive processing, neurophysiological markers, and intervention-oriented domains. This dispersion is consistent with the broader challenge of studying mental workload and cognitive demand, where no single physiological or behavioural measure can fully capture the construct across tasks and contexts [54].

The included studies used diverse objective and semi-objective indicators, including heart rate, HRV, EDA, cortisol, EEG, ERP, fNIRS, eye-tracking, pupil diameter, sleep- and wearable-derived measures, cognitive-performance tasks, behavioural indicators, and in-game performance metrics. This methodological breadth indicates that esports research has moved beyond purely subjective descriptions of gaming experience, but it also highlights the absence of a shared measurement framework for cognitive overload. Related states are currently inferred from different combinations of physiological arousal, cognitive performance, attentional behaviour, sleep disruption, emotional strain, and gameplay outcomes, which complicates comparison across studies and limits cumulative interpretation [54,55].

A further principal finding is that overload-related processes appear to be strongly context-dependent. The demands of esports vary across game genres, competitive settings, and player levels. Multiplayer online battle arena games, tactical shooters, fighting games, sports simulations, and aiming-based tasks do not impose identical cognitive or psychophysiological demands, and genre classification itself remains a methodological challenge in psychological games research [56]. Similarly, laboratory tasks, ranked online matches, collegiate tournaments, professional competition, and longitudinal monitoring protocols differ in ecological validity and experimental control. This heterogeneity complicates direct comparison across studies, while also illustrating the value of esports as a naturalistic context for studying behaviour in interactive digital systems [57].

Thus, the main contribution of this review is not to identify a dominant marker of cognitive overload, but to demonstrate that the current evidence base lacks construct-specific convergence.

### 4.2. Interpretation in Relation to Cognitive Overload

The findings suggest that cognitive overload in esports is best understood as a dynamic and multidimensional state rather than a discrete outcome. This interpretation is consistent with contemporary workload research, which treats mental workload as context-sensitive and measurable only through a combination of subjective, behavioural, physiological, and neurophysiological indicators [55,58]. In competitive play, overload may emerge when players must sustain attention, process rapidly changing information, coordinate perception and action, regulate emotion, communicate with teammates, and maintain performance under time pressure or social evaluation. These demands are likely to fluctuate across match phases, critical in-game events, opponent behaviour, perceived match importance, fatigue, and recent success or failure.

This interpretation also aligns with research on esports as a fast-paced, computer-mediated team environment. The game system does not merely present a task; it continuously generates feedback, uncertainty, constraints, opportunities, and consequences. Players interact with the interface, game mechanics, teammates, opponents, and ranking or tournament systems simultaneously, while team coordination depends on shared awareness, role interdependence, communication, and rapid adaptation to a changing virtual environment [59,60]. Cognitive overload therefore reflects not only the amount of information presented to the player, but also the coordination required to regulate attention, emotion, action, and performance within a changing digital performance ecology.

An important conceptual issue concerns the distinction between cognitive overload and competitive stress. These constructs are likely to interact in esports, but they should not be treated as equivalent. Cognitive overload refers to the relation between task demands and available cognitive-regulatory resources, whereas competitive stress reflects the appraisal of a situation as challenging, threatening, consequential, or socially evaluative. Experimental and psychophysiological work shows that mental workload and acute stress can produce partly overlapping but distinguishable physiological signatures, while acute stress can impair executive functions relevant to competitive performance, including working memory and cognitive flexibility [61,62]. The same in-game event may therefore increase both information-processing demands and stress responses, making markers such as HR, HRV, pupil diameter, EDA, or cortisol difficult to interpret as direct indicators of overload.

The review also indicates that overload-related states in esports are often expressed through concepts familiar to players yet not fully integrated into formal research models. Terms such as tilt, pressure, fatigue, and performance decline capture experiences associated with emotional disruption, impaired decision-making, and reduced ability to recover from negative in-game events. Recent work on tilt in esports suggests that such community-derived concepts can identify psychologically meaningful states that affect both emotion regulation and gameplay behaviour [63]. These concepts are therefore relevant to cognitive-overload research but require clearer operational definitions to support cumulative theory-building.

A key implication is that cognitive overload should not be reduced to a single marker. Elevated HR, reduced HRV, increased pupil diameter, altered EEG activity, poorer reaction time, reduced sleep quality, or declining in-game performance may each capture one component of overload-related functioning. However, physiological indicators vary in sensitivity across tasks, and pupil-based or autonomic responses may reflect cognitive effort, arousal, emotional activation, or compensatory regulation rather than overload alone [58,64]. A more informative approach is to conceptualize cognitive overload as an integrative construct linking subjective experience, physiological regulation, cognitive efficiency, attentional behaviour, and performance change.

### 4.3. Measurement and Methodological Implications

The current evidence base demonstrates considerable methodological diversity. This diversity is valuable, as it shows that esports can be examined through multimodal approaches combining psychophysiology, neurophysiology, eye-tracking, sleep monitoring, cognitive testing, machine learning, and in-game telemetry [65,66]. However, the absence of standardized protocols and construct-specific measurement models limits comparability across studies and constrains the development of cumulative theory.

A second priority concerns multimodal integration. Many studies measured one or two indicators, whereas relatively few integrated physiological, cognitive, behavioural, and in-game data into a coherent model. Esports is particularly suitable for such integration, as it naturally generates rich behavioural traces, repeated performance events, and temporally structured gameplay data. Time-synchronized physiological markers, in-game events, player actions, communication patterns, and match outcomes could make it possible to identify when overload emerges, how it develops, and which indicators are most sensitive to performance-relevant disruption [66,67].

A third implication relates to ecological validity. Laboratory studies offer experimental control, but may not fully capture the social, strategic, emotional, and ranking-related pressures of real competitive play. Field and tournament studies provide more naturalistic conditions, although they often reduce control over confounding variables. This trade-off is consistent with broader concerns in game-based research, where games provide rich and ecologically meaningful environments but also introduce systemic complexity, variance, and framing effects that may threaten validity [68]. The most informative future designs may therefore combine controlled tasks to isolate mechanisms with ecologically valid protocols to examine whether those mechanisms generalize to ranked, collegiate, semi-professional, or professional competitive settings.

Finally, game genre and competitive level should be treated as core methodological variables. Aggregating esports titles into a single category may obscure important differences in cognitive demand, while treating ranked, collegiate, semi-professional, professional, and elite players as interchangeable may obscure differences in expertise, pressure exposure, and regulatory capacity. Empirical comparisons between FPS and MOBA players indicate that genre may be associated with different cognitive profiles, including differences in sustained attention, reaction time, and inhibitory control [21]. Genre specificity also extends beyond cognition, as FPS, MOBA, and other game types may impose different motor and kinematic demands during play [69], while discrete gameplay sessions may alter both cognitive and physiological outcomes [70]. Expertise may further influence how efficiently players allocate attention, anticipate game events, regulate arousal, recover from errors, and maintain performance under pressure. Meta-analytic evidence suggests that esports experts differ from amateurs particularly in spatial cognition and attention [25], and game-specific work in League of Legends indicates that expert players may show stronger visuospatial working memory and attentional control than regular players or non-players [71]. Higher-level players may therefore tolerate greater workload before overload-related disruption appears, but they may also experience stronger competitive demands, training volume, social evaluation, and performance consequences. Studies should therefore report the game title, genre, player role where relevant, competitive level, basis for competitive classification, match format, task type, and performance metric in sufficient detail. Such reporting would help distinguish general properties of competitive digital performance from genre-specific and expertise-related mechanisms, as different game genres and player levels may be associated with different cognitive functions, interaction patterns, psychophysiological responses, and overload thresholds [72,73]. These differences also have practical implications for assessment protocols. A universal cognitive or psychophysiological battery may overlook genre-specific demands, since the markers most relevant to an FPS clutch situation, a MOBA team fight, a racing task, or a sport-simulation match may differ substantially. Future studies may therefore benefit from genre-sensitive assessment batteries that combine shared core measures, such as workload, arousal, fatigue, and performance stability, with task-specific indicators reflecting the cognitive and interaction demands of the game genre under investigation.

### 4.4. Practical Interpretation of Marker Families

The methodological heterogeneity of the reviewed studies also has practical implications for the interpretation of objective and semi-objective markers. Although HR, HRV, EDA, cortisol, EEG/ERP, fNIRS, eye-tracking, pupillometry, sleep-related indicators, cognitive-performance tests, and in-game metrics were all used to study overload-related processes, these markers should not be treated as interchangeable indicators of cognitive overload. Each marker captures a different level of the demand–resource–regulation process and differs in temporal sensitivity, ecological validity, feasibility, and specificity. Therefore, marker selection should depend on the research question, game genre, study context, player population, and whether the aim is to capture acute arousal, sustained fatigue, attentional allocation, neural activity, recovery status, or performance disruption. Table 4 summarizes the relative strengths, limitations, and practical applicability of the main marker families identified in this review.

### 4.5. Potential Implications for Research and Applied Esports Contexts

Given the scoping nature of this review and the absence of formal critical appraisal, the findings should be interpreted as a descriptive map of emerging evidence rather than as evidence-based recommendations for practice. The included studies suggest that cognitive and psychophysiological regulation may be relevant to esports performance, but the strength, consistency, and methodological quality of this evidence were not formally evaluated. Therefore, any applied use of markers such as HRV, sleep measures, eye-tracking, cognitive-performance tests, or in-game metrics should be considered exploratory and context-dependent.

For esports athletes and coaches, the mapped evidence suggests that performance support may need to consider factors beyond mechanical skill and game strategy, including recovery, sleep, arousal regulation, attentional control, and workload management. However, as the present review did not appraise the methodological quality of included studies, these observations should be treated as hypothesis-generating rather than prescriptive. Markers such as HRV, sleep measures, eye-tracking, or in-game performance data may be useful in future monitoring frameworks, but their applied value requires further validation in ecologically valid esports settings.

Coping and regulation strategies represent an emerging, but still preliminary, area of esports research. Existing work in esports has examined stressors and coping strategies, sleep-focused interventions, arousal reappraisal, mindset interventions, and biofeedback-based performance training [74,75,76,77]. These approaches target different components of overload-related functioning, including arousal regulation, attentional control, sleep quality, cognitive readiness, and performance under pressure. However, the available studies vary in design, sample size, competitive level, intervention duration, and outcome selection. Consequently, the present review cannot determine which strategies are effective, for whom, or under which competitive conditions. Current evidence therefore does not support a single universal strategy, but indicates that individualized, context-sensitive monitoring combining subjective feedback with objective indicators may be a useful direction for future applied research rather than a definitive recommendation for practice.

For researchers in human–computer interaction and cyberpsychology, esports provides a useful model of performance in interactive digital systems. Unlike many laboratory tasks, esports involves real-time decision-making, repeated feedback, measurable outcomes, social coordination, and motivationally meaningful consequences. These characteristics make esports relevant not only for competitive gaming research, but also for broader questions concerning cognitive workload, adaptive interfaces, affective computing, digital performance monitoring, and human behaviour under pressure.

For technology development, the findings suggest a longer-term research opportunity for adaptive systems aimed at estimating overload-related states in real time. Such systems could integrate physiological sensing, eye-tracking, gameplay telemetry, and behavioural modelling to identify fatigue, stress, or performance decline. However, implementation would require careful attention to privacy, data ownership, player surveillance, and the potential misuse of psychophysiological data in competitive environments, particularly when affective or physiological data are used to infer internal states [78,79].

The intervention-oriented evidence should be interpreted cautiously as the included studies differed substantially in design, sample size, outcome selection, and ecological validity. At present, sleep and recovery-oriented approaches appear to have the strongest conceptual relevance for cognitive-overload research as they target fatigue, readiness, mood, and restoration of cognitive and regulatory resources, with esports-specific sleep intervention work supporting their practical relevance [74]. Exercise and general lifestyle interventions may also support recovery and psychophysiological regulation, with dual-review evidence suggesting that exercise may benefit cognitive functions relevant to esports performance, although esports-specific intervention effects remain insufficiently established [80]. Biofeedback, mindset, reappraisal, and emotion-regulation approaches are promising as they directly address arousal control, stress appraisal, and self-regulation during performance; however, evidence from sport biofeedback and esports-specific reappraisal studies indicates that these approaches still require replication in larger, genre-sensitive, and ecologically valid samples [77,81]. Nutrition, caffeine, and other performance-enhancement strategies should be interpreted with particular caution, as esports studies show mixed and context-dependent effects: energy drink consumption did not clearly improve cognitive or physical performance in elite League of Legends players, whereas caffeine improved shooting performance and reaction time in FPS players under controlled conditions [82,83]. The current literature does not yet support one dominant intervention for reducing cognitive overload in esports. Rather, it suggests that future work should compare recovery, regulation, training, and enhancement strategies using multimodal markers and game-specific performance outcomes.

From an applied perspective, the findings suggest that esports practitioners, coaches, sport psychologists, and performance scientists should avoid relying on a single marker of cognitive overload. Monitoring strategies should combine game-specific performance information with selected physiological, ocular, subjective, and recovery-related indicators, depending on the genre and competitive context. For example, HR/HRV and sleep- or wearable-derived recovery indicators may be useful for tracking readiness and autonomic strain across training blocks, while eye-tracking, pupillometry, EDA/GSR, and in-game telemetry may help identify acute pressure moments, attentional disruption, or performance breakdowns during specific gameplay events. Applied monitoring should therefore be time-synchronized with match phases, errors, clutch situations, team fights, or other critical in-game moments rather than interpreted only as session-level averages, as physiological monitoring and synchronized sensor–telemetry systems have been proposed as important tools for esports performance assessment and training analytics [84,85]. Recovery management should also be treated as part of overload prevention, particularly when players are exposed to prolonged training, poor sleep, repeated competition, or high emotional pressure.

### 4.6. Limitations

Several limitations should be acknowledged.

First, consistent with the purpose of a scoping review, no formal risk-of-bias assessment or methodological quality appraisal was conducted. The findings should therefore be interpreted as an evidence map rather than as a judgement about the strength, quality, or effectiveness of specific markers, interventions, or theoretical claims. This limitation is important as the included studies varied substantially in design, sample size, ecological validity, competitive level, and measurement approach. Consequently, exploratory laboratory studies, small-sample psychophysiological protocols, field-based observations, intervention-oriented studies, and larger empirical datasets were not ranked according to methodological robustness. The review identifies what has been studied and how cognitive-overload-related constructs have been measured, but it does not establish the certainty of evidence supporting specific monitoring tools, intervention strategies, or applied recommendations. Second, the review was restricted to peer-reviewed, English-language, full-text journal articles. This criterion was applied to ensure a consistent minimum level of methodological reporting, scientific scrutiny, and comparability across included sources. However, this decision also narrowed the scope of the evidence map and may have excluded relevant work disseminated through conference proceedings, preprints, dissertations, technical reports, datasets, or non-English publications. This limitation is particularly important in esports, human–computer interaction, applied computing, and performance-science research, where conference publications and rapidly emerging technical or empirical work can make substantial contributions to the field. In addition, although the search included Scopus, PubMed, Web of Science, and EBSCOhost, other databases relevant to psychology, sport science, engineering, and human–computer interaction, such as PsycINFO, SPORTDiscus, IEEE Xplore, and ACM Digital Library, were not searched separately. Therefore, some relevant studies published in psychology-, sport-, computing-, or HCI-oriented venues may have been missed. This limitation should be considered when interpreting the completeness of the evidence map. Third, the included studies were highly heterogeneous in terms of design, player population, competitive level, game title, game genre, setting, measurement protocol, and outcome definition. This heterogeneity is informative for mapping the field, but it limits direct comparison across studies and precludes quantitative synthesis.

Fourth, competitive status was based on author-reported information such as official rank, tournament participation, collegiate membership, semi-professional or professional affiliation, elite classification, or structured competitive practice. Although these criteria were appropriate for a scoping review, they were not standardized across studies and may have captured different levels of competitive engagement.

Fifth, cognitive overload was rarely defined directly in the included literature and was usually inferred through adjacent constructs such as mental workload, stress, arousal, fatigue, sleepiness, tilt, attentional disruption, or performance decline. As a result, the present synthesis necessarily treats cognitive overload as an umbrella construct rather than as a consistently measured outcome.

Another, esports-specific, limitation concerns the demographic composition of the included samples. Many studies involved predominantly male participants, which limits the external validity of the evidence base for female esports athletes and more diverse competitive populations. This imbalance should not be interpreted solely as a methodological weakness of individual studies, as it also reflects the broader structure of competitive esports, where male players are often more visible, more represented in organized competitive settings, and more accessible for recruitment. Nevertheless, the resulting evidence base may underrepresent sex- and gender-related differences in cognitive regulation, stress responses, recovery, social evaluation, and performance pressure. Future research should therefore make greater efforts to recruit, report, and analyse more diverse esports samples, including female players and underrepresented competitive groups.

Funding sources of the included studies were not systematically charted because this review focused on study characteristics, populations, markers, outcomes, and overload-related domains rather than funding patterns across the evidence base.

Finally, the conclusions are limited by the methodological characteristics of the available evidence. Many studies relied on small, male-dominated, or highly specific samples, and relatively few integrated multiple modalities such as physiological markers, eye-tracking, sleep measures, cognitive tests, and in-game telemetry within the same design. Moreover, several studies used laboratory or simulated esports tasks, which may not fully reproduce the social, strategic, emotional, and ranking-related demands of real competitive play.

### 4.7. Future Research Directions

Future research should move toward clearer conceptual and operational models of cognitive overload in esports. Studies should explicitly define the construct under investigation, such as mental workload, stress, arousal, fatigue, tilt, sleep-related impairment, attentional disruption, or performance decline, and justify how the selected markers reflect that construct. These constructs should not be treated as interchangeable: mental workload refers to task demands and resource allocation; stress and arousal reflect responses to perceived challenge or threat; fatigue and sleep loss may reduce cognitive efficiency over time; and performance decline represents a behavioural consequence that may arise from several interacting mechanisms [86]. Greater terminological precision would improve comparability across studies and support cumulative theory-building.

Longitudinal and event-based designs are particularly needed. Rather than relying primarily on pre–post comparisons or aggregate match-level outcomes, future studies should examine how cognitive and psychophysiological states fluctuate across match phases, critical events, role-specific demands, and periods of sustained practice or competition. Time-synchronized physiological, behavioural, and in-game data could provide a more precise understanding of when overload emerges and how it affects performance.

More research is also needed in ecologically valid competitive settings. Laboratory protocols remain valuable, but findings should be tested in ranked play, team practice, collegiate competition, semi-professional environments, and professional tournaments.

Studies should also recruit more diverse samples, including female players, mixed-gender teams, different age groups, and players across a wider range of competitive levels.

Future work should examine game genre more systematically. Different esports titles may require different profiles of attention, decision-making, coordination, and emotional regulation. Comparative studies across genres could clarify whether cognitive overload has shared features across esports or whether it is primarily task- and genre-specific.

Intervention research should also be expanded. Biofeedback, sleep optimization, exercise, psychological skills training, reappraisal, recovery protocols, and adaptive performance-monitoring systems all represent promising directions. Future interventions should be tested with adequate sample sizes, longer follow-up periods, ecologically meaningful performance outcomes, and multimodal measurement strategies.

A further conceptual priority is to avoid interpreting psychophysiological activation as a direct proxy for cognitive overload without considering the competitive meaning of the task. Stress may contribute to overload by consuming attentional resources, increasing intrusive thoughts, narrowing attentional focus, or impairing emotion regulation. Conversely, a high cognitive load may itself become stressful when players perceive loss of control over the game state or failure to meet performance expectations. Disentangling these directions of influence is particularly challenging in esports, where cognitive demand and competitive evaluation are embedded in the same digital event. This distinction represents a central conceptual and methodological priority for research on overload in competitive human–computer interaction.

## 5. Conclusions

This scoping review suggests that cognitive overload in esports should be understood not as a single measurable outcome, but as a cognitive, affective, behavioural, and psychophysiological state emerging during intensive interaction with complex digital systems. Competitive esports exposes players to rapidly changing perceptual information, continuous action–feedback loops, social evaluation, and performance-contingent consequences. In this context, overload may arise from the coupling of attentional control, executive regulation, affective appraisal, sensorimotor coordination, and autonomic activation.

The reviewed evidence indicates that esports research has begun to capture these processes through cardiovascular and autonomic indices, ocular measures, EEG/ERP, fNIRS, sleep-related indicators, cognitive-performance tests, and in-game behavioural data. However, these measures have rarely been integrated into unified, time-sensitive models of how overload-related processes develop during competitive digital play. The available evidence therefore remains scattered, multimodal, and conceptually heterogeneous. Rather than establishing a consolidated model of cognitive overload in esports, the present review provides a structured synthesis of how adjacent constructs, markers, and outcomes have been studied across the field.

A central conclusion is that psychophysiological activation in esports should not be interpreted as a direct proxy for cognitive overload. Increased arousal may reflect cognitive effort, competitive stress, emotional reactivity, fatigue, or adaptive mobilization for performance. Future work should therefore distinguish more clearly between workload, stress appraisal, fatigue, tilt, and performance decline while also examining how these processes interact over time. Multimodal, time-synchronized approaches combining neural, physiological, ocular, behavioural, and in-game data appear especially promising for this purpose, provided that they are interpreted in relation to game genre, competitive context, player expertise, and ecological validity.

More broadly, esports offers a naturalistic model for studying cognition and behaviour under demanding computer-mediated conditions. It provides a rare environment in which complex human performance can be examined through both ecological competitive contexts and objective digital traces. Advancing this field will require theory-informed, genre-sensitive, and multimodal models of overload that account for the dynamic interaction between the player and the digital performance environment. Such work may help clarify when a high workload reflects adaptive engagement, when it becomes inefficient or disruptive, and which monitoring or recovery strategies are most appropriate for supporting performance and wellbeing in competitive esports.

## Figures and Tables

**Table 1:** Population, Concept, and Context framework for the review.

PCC Element	Definition
Population	Ranked, competitive, collegiate, semi-professional, professional, or elite esports players
Concept	Cognitive overload and related constructs, including cognitive load, mental workload, fatigue, stress, arousal, attention, coping, recovery, psychophysiological regulation, and performance
Context	Esports or competitive gaming contexts, including laboratory, training, online, tournament, and field settings

**Table 2:** Summary of the Studies Included in the Review.

Characteristic	Summary
Publication period	The included studies were published between 2016 and 2026. Most studies were published from 2021 onward (*n* = 42; 87.5%), with the highest number of publications recorded in 2024 (*n* = 15; 31.3%).
Geographical distribution	United States (*n* = 7); Japan (*n* = 6); China (*n* = 7); Germany (*n* = 4); Australia (*n* = 3); Spain (*n* = 3); other, multinational, or not clearly specified contexts (*n* = 18).
Dominant study design	experimental/intervention (*n* = 18); cross-sectional/comparative (*n* = 14); observational field/tournament (*n* = 10); longitudinal/repeated monitoring (*n* = 3); dataset/machine-learning/secondary analysis (*n* = 1); other/mixed (*n* = 2).
Player population/competitive level	professional/elite/high-skilled (*n* = 26); ranked/competitive/semi-professional (*n* = 17); recreational competitive (*n* = 8); collegiate/university (*n* = 7). Categories are non-mutually exclusive.
Game genres and main titles	Genres: FPS/tactical or hero shooter (*n* = 23); MOBA (*n* = 22); mixed/multiple genres (*n* = 8); sports simulation/electronic football (*n* = 5); fighting game (*n* = 4); Rocket League/vehicular soccer (*n* = 3); FPS-like aiming task (*n* = 3). Most frequent titles/contexts: League of Legends (*n* = 19); Counter-Strike/CS:GO (*n* = 13); FIFA/eFootball/virtual football (*n* = 5); Overwatch (*n* = 5); Valorant (*n* = 4); Rocket League (*n* = 3); Dota 2 (*n* = 2). Genre categories are non-mutually exclusive.
Performance and cognitive outcomes	in-game/task performance metrics (*n* = 37); cognitive-performance tests (*n* = 19).
Physiological, psychophysiological, neurophysiological, and sleep-related markers	heart rate (HR)/cardiovascular measures (*n* = 14); heart rate variability (HRV)/autonomic regulation (*n* = 11); eye-tracking/gaze/visual behaviour (*n* = 9); sleep/actigraphy/wearables (*n* = 8); metabolic/respiratory/energy expenditure (*n* = 6); electroencephalography (EEG),/ event-related potential (ERP) (*n* = 6); cortisol/endocrine markers (*n* = 6); pupil diameter/pupillometry (*n* = 4); electrodermal activity/galvanic skin response (EDA/GSR)/skin conductance (*n* = 3); functional near-infrared spectroscopy (fNIRS)/cerebral hemodynamics (*n* = 1). Categories are non-mutually exclusive.

**Table 3:** Evidence map of cognitive-overload-related domains, common markers, and typical outcomes.

Evidence Domain	n Studies	Common Markers/Measures	Typical Focus/Outcomes
In-game performance, practice, and expertise outcomes	37	Match outcomes, game logs/statistics, rank/matchmaking rating (MMR), task accuracy/time, expert–novice classification	Performance prediction, expertise differences, practice behaviour, win/loss, and task performance
Stress, arousal, and autonomic regulation	29	HR, HRV, blood pressure, pulse-wave velocity, cortisol, anxiety/stress measures	Competition responses, victory/defeat, pressure, and sympathetic–parasympathetic regulation
Cardiometabolic and physiological load	24	Energy expenditure, VO_2_/VCO_2_, respiratory exchange ratio, HR/HRV, blood pressure, glucose/lactate	Physiological load during gameplay and cardiovascular/metabolic responses to competitive play
Mental workload, cognitive load, and performance decline	20	HRV, EDA/GSR, workload ratings, machine-learning features, behavioural/performance-decline labels	Mental workload classification, tilt/performance decline detection, flow/anxiety relationships
Sleep, fatigue, and recovery	19	Actigraphy/wearables, Oura/Somnofy, sleep diaries, HRV, PVT/reaction time, sleepiness/fatigue ratings	Sleep duration/quality, sleep timing, sleep deprivation, recovery, mood, and cognitive performance
Visual attention, gaze, and perceptual-cognitive processing	15	Eye-tracking, fixation metrics, gaze distribution, scan patterns, pupil diameter, visual search, multiple-object tracking, visual short-term memory	Expert–novice differences, gaze strategies, visual memory, and game-specific attentional processing
Neurophysiological and cortical markers	15	EEG, ERP/P300, spectral power, resting-state EEG complexity, fNIRS/cerebral hemoglobin	Expertise classification, neural predictors of performance, cortical activation, and cognitive control
Intervention, coping, and regulation strategies	12	Biofeedback, reappraisal/mindset, exercise, sleep education/counselling, energy drink, nutrition/lifestyle	Performance optimization, anxiety/appraisals, sleep/recovery, HRV/cerebral oxygenation, and coping

**Table 4:** Strengths, limitations, and esports-specific applicability of marker families used to assess cognitive-overload-related processes.

Marker Family	Strength	Limitation	Esports-Specific Applicability
HR/HRV	Easy to collect; useful for autonomic activation, arousal, and recovery monitoring.	Non-specific; affected by posture, caffeine, sleep, fitness, emotion, and baseline differences.	Useful during scrims, ranked sessions, tournaments, and recovery monitoring when synchronized with match events.
EDA/GSR	Sensitive to short-term sympathetic arousal and event-related activation.	Cannot distinguish effort, stress, excitement, or frustration without context.	Useful for clutch moments, mistakes, team fights, overtime phases, and high-pressure rounds.
Cortisol	Captures slower endocrine responses to competition-related stress.	Low temporal resolution; affected by circadian rhythm, sleep, food intake, and sampling protocol.	Better suited to pre–post tournament or prolonged competition studies than real-time gameplay monitoring.
EEG/ERP/fNIRS	Closer to neural processes involved in attention, cognitive control, expertise, and workload.	Technically demanding; sensitive to artifacts; usually requires controlled conditions.	Useful for mechanistic studies of expertise, attention, inhibition, and workload in standardized esports tasks.
Eye-tracking/pupillometry	Captures gaze allocation, visual search, attentional focus, and effort-related pupil responses.	Strongly affected by interface, lighting, calibration, screen layout, and game genre.	Highly relevant for FPS, MOBA, aiming, racing, and visual-search-heavy tasks.
Sleep/wearable recovery indicators	Useful for fatigue, readiness, sleep timing, and recovery status.	Does not directly measure overload during gameplay; device accuracy varies.	Helps explain why similar gameplay demands may become manageable on some days and overload-inducing on others.
Cognitive-performance tests	Assess attention, inhibition, reaction time, working memory, and processing efficiency.	Ecological validity depends on similarity to game-specific demands; learning effects may occur.	Useful for player profiling, fatigue monitoring, and pre–post assessment when paired with game-relevant tasks.
In-game telemetry	Ecological, scalable, and temporally precise; directly reflects gameplay behaviour.	Difficult to compare across games; affected by role, team dynamics, opponent level, strategy, and patches.	Essential for time-synchronized esports models linking overload markers with match phase, errors, clutch events, and performance breakdowns.
Subjective ratings	Capture perceived workload, fatigue, stress, frustration, confidence, and affective state.	Vulnerable to recall bias, social desirability, and mismatch with physiological activation.	Useful for connecting objective markers with player experience after matches, rounds, or standardized gameplay segments.
Multimodal combinations	Integrate physiological, ocular, cognitive, subjective, sleep-related, and in-game data.	Require synchronization, larger datasets, and clear theoretical assumptions.	Most suitable for future esports overload models as no single marker can capture workload, stress, fatigue, tilt, and performance decline alone.

## Data Availability

The original contributions presented in this study are included in the article/Supplementary Material. Further inquiries can be directed to the corresponding authors.

## Supplementary Materials

The following supporting information can be downloaded at: https://www.mdpi.com/article/10.3390/medsci14030395/s1. (References [87,88,89,90,91,92,93,94,95,96,97,98,99,100,101,102,103,104,105,106,107,108,109,110,111,112,113,114,115,116,117,118,119,120,121,122,123,124] are cited in the Supplementary Materials). Supplementary File S1. Search strings. Supplementary Table S1. Characteristics of included studies. Supplementary Table S2. Evidence map of cognitive-overload-related domains, markers, and outcomes. Supplementary Table S3. Characteristics of excluded studies. Supplementary Table S4. List of EBSCO bases searched. Supplementary File S2. Preferred Reporting Items for Systematic reviews and Meta-Analyses extension for Scoping Reviews (PRISMA-ScR) Checklist.
